# miR193a-5p Mediated ZNF746 and c-Myc Signaling Axis Is Critically Involved in Morusin Induced Apoptosis in Colorectal Cancer Cells

**DOI:** 10.3390/cells10082065

**Published:** 2021-08-12

**Authors:** Woon-Yi Park, Hyo-Jung Lee, Deok-Yong Sim, Eunji Im, Ji-Eon Park, Chi-Hoon Ahn, Bum-Sang Shim, Sung-Hoon Kim

**Affiliations:** Department of Korean Pathology, College of Korean Medicine, Kyung Hee University, Seoul 02447, Korea; wy1319@naver.com (W.-Y.P.); hyonice77@naver.com (H.-J.L.); simdy0821@naver.com (D.-Y.S.); ji4137@naver.com (E.I.); wdnk77@naver.com (J.-E.P.); ach2565@naver.com (C.-H.A.)

**Keywords:** morusin, colorectal cancer, apoptosis, ZNF746/c-Myc, miR-193a-5p

## Abstract

Novel target therapy is on the spotlight for effective cancer therapy. Hence, in the present study, the underlying apoptotic mechanism of Morusin was explored in association with miR193a-5p mediated ZNF746/c-Myc signaling axis in colorectal cancer cells (CRCs). Herein, Morusin reduced the viability and the number of colonies in HCT116 and SW480 CRCs. Additionally, Morusin increased sub-G1 population, cleavages of poly (ADP-ribose) polymerase (PARP) and caspase-3 and inhibited the expression of zinc finger protein 746 (ZNF746) and c-Myc in HCT116 and SW480 cells. Conversely, overexpression of ZNF746 suppressed the ability of Morusin to abrogate the expression of c-Myc in HCT116 cells, as ZNF746 enhanced the stability of c-Myc via their direct binding through nuclear colocalization in HCT116 cells by immunofluorescence and immunoprecipitation. Notably, Morusin upregulated miR193a-5p as a tumor suppressor, while miR193a-5p inhibitor masked the ability of Morusin to reduce the expression of ZNF746, c-Myc, and pro-PARP in HCT116 cells. To our knowledge, these findings provide the novel insight on miR193a-5p mediated inhibition of ZNF746/c-Myc signaling in Morusin induced apoptosis in CRCs.

## 1. Introduction

Colorectal cancer (CRC) is reported as the fourth most prevalent cancer in women and the third most common cancer in men worldwide [1,2]. Despite modern medicine, approximately 50% of CRC patients have experienced tumor recurrence, and their overall mortality rate is estimated up to approximately 40% [3]. Though chemotherapy, mainly with 5-FU, oxaliplatin (FOLFOX), Irinotecan and Cetuximab, radiotherapy and surgery. have been utilized for CRC treatment for years, recently molecular target therapy is of interest for EGFR, RAS, and VEGF [4,5].

It is well documented that c-Myc is a biomarker for poor prognosis of CRC patients, as it is a transcription factor that is critically associated with cell growth, cell adhesion, proliferation, and apoptosis [6]. Hence, to target c-Myc has been considered a good strategy for prevention or treatment of CRCs [7]. Additionally, ZNF746, with a C2HC/C2H2 type zinc finger protein at its C terminus [8,9] and a Kruppel-associated box at its N terminus [10,11], is known to act as a suppressor of PPAR gamma [12], regulate Parkin as a ubiquitin E3 ligase [13], and enhance the stability of c-Myc [1]. Additionally, accumulating evidence reveals that many miRNAs are critically involved in cancer biology as oncogenes or tumor suppressors [14,15,16].

Additionally, some natural compounds such as quercetin [17], ursolic acid [18], and curcumin [19] are being considered for combination therapy with classical anticancer agents. In the same line, Morusin, one of the prenylated flavonoids [20,21,22] derived from the root bark of *Morus alba*, is known to have anti-microbial [23], anti-inflammatory [24], and antitumor [3,25,26] effects. Nonetheless, its underlying molecular mechanism remains unclear in CRCs to date. Thus, in the present study, the molecular mechanism of Morusin was explored in CRCs, targeting c-Myc and ZNF746 signaling mediated by miRNA 193a-5p as a tumor suppressor.

## 2. Materials and Methods

### 2.1. Cell Culture

Human colorectal HCT116 (ATCC^®^ CCL-247), SW480 (ATCC^®^ CCL-228) cancer cell lines supplied from American Type Culture Collection (ATCC) were maintained in RPMI1640 with 1% antibiotic and 10% FBS (Welgene, Gyeongsan, Korea) in Forma 320 CO_2_ incubator (Marshal Scientific Company, Hampton, NH, USA). All experiments were conducted in the 75–80% confluence of the cells.

### 2.2. Cell Viability Assay

Based on Jung et al.’s paper [1], cell viability assay was conducted in HCT116 and SW480 CRCs by using MTT assay. Briefly, HCT116 and SW480 cells (1 × 10^4^ cells/well) were exposed to Morusin (0, 2.5, 5, and 10 μM) for 24 h and incubated with MTT (1 mg/mL) (Sigma-Aldrich, Saint Louis, MO, USA) for 2 h. The viability was calculated with optical density (OD) values as a percentage of viable cells in Morusin treated group versus untreated control. All assays were conducted in independent triplicates.

### 2.3. Colony Formation Assay

HCT116 and SW480 cells (3 × 10^3^/well) exposed to Morusin (0, 2.5, and 5 μM) for 24 h were distributed onto 6-well plates for a week. The cells were washed PBS, fixed, and stained with Diff quick solution (Sysmex, Kobe, Japan). Then, the colonies were counted under inverted microscope.

### 2.4. Cell Cycle Analysis

HCT116 and SW480 cells (2 × 10^5^ cells/mL) exposed to Morusin (0, 2.5, or 5 μM) for 24 h were incubated with RNase A (10 mg/mL) for 1 h at 37 °C and stained with propidium iodide (50 μg/mL) in dark. The stained cells were analyzed for the DNA content by FACS Calibur with CellQuest Software.

### 2.5. Cycloheximide Assay

HCT116 and SW480 cells treated by Morusin (5 μM) for 24 h were exposed to 50 μg/mL cycloheximide for various times (0, 15, 30, and 60 min) before Western blotting.

### 2.6. Western Blotting

Based on Kim et al.’s paper [12], HCT116 and SW480 cells (1 × 10^6^ cells/mL) were exposed to Morusin (0, 2.5, or 5 μM) of for 24 h, The supernatants were collected and quantified for protein concentration by using RC DC protein assay kit (Bio-Rad, Hercules, CA, USA), The protein samples (30 μg of protein from cells) were separated on 4–12% NuPAGE Bis–Tris gels (Novex, Carlsbad, CA, USA) and ECL transfer membrane for detection with antibodies for PARP (#9542), pro-caspase-3 (#9662), cleaved caspase-3 (#9664), c-Myc (Y69) and ZNF746 (LS-B8045), Bcl-2 (SC-492), Bcl-xL (SC-8392), and β-actin (A2228) (Sigma, St. Louis, MO, USA). Proteins were detected using the ECL system (Amersham Pharmacia Biotech INC, District of Columbia, MO, USA). Densitometric analysis was performed using ImageJ software.

### 2.7. TUNEL Assay

Based on Park et al.’s paper [6], to detect cell death, HCT116 or SW480 cells were treated with Morusin (5 μM) for 24 h, and incubated with TUNEL assay mixture for 60 min by using the DeadEnd™ Fluorometric TUNEL system kit (Promega, Madison, WI, USA). Then, TUNEL-stained cells were visualized by a Delta Vision imaging system (Applied Precision, Issaquah, WA, USA).

### 2.8. RT-qPCR Analysis

Based on Jung et al.’s paper [1], total RNA from HCT116 cells exposed to Morusin (5 μM) was isolated by QIAzol (Invitrogen, Carlsbad, CA, USA) and synthesized with oligo dT (Bioneer, Daejeon, Korea) and M-MLV reverse transcriptase (Enzynomics, Daejeon, Korea), followed by qRT-PCR analysis by using Light cyclerTM (Roche, Basel, Switzerland) for miR193a-5p.

### 2.9. RNA Interference

HCT116 cells were transfected with miR-193a-5p mimic, miR-193a-5p inhibitor Sequence (5′-3′):UGGGUCUUUGCGGGCGAGAUGA, miR-Con, and miR-Con inhibitor (200 nM) (Bioneer, Daejeon, Korea), or Flag-ZNF746 plasmids by using X-tremeGENE HP DNA Transfection Reagent (Roche, Basel, Switzerland) for next experiments.

### 2.10. Co-Immunoprecipitation

HCT116 cells exposed to Morusin (5 μM) for 24 h were lyzed in lysis buffer and immunoprecipitated with ZNF746 antibody or normal immunoglobulin G antibody, then protein A/G sepharose beads (Santa Cruz Biotechnology, Santa Cruz, CA, USA). The precipitated proteins were subjected to immunoblotting with the antibodies of ZNF746 and c-Myc.

### 2.11. Immunofluorescence

HCT116 cells exposed to Morusin (5 μM) for 24 h were fixed with 4% formaldehyde and then permeabilized in 0.1% Triton X-100. The fixed cells were incubated with primary antibodies of ZNF746, c-Myc (Cell signaling, Boston, MA, USA) and then incubated with Alexa Fluor 546 goat rabbit-IgG antibody (Life technologies, Waltham, MA, USA) (1:1000) for 1 h. Finally, the nuclei of the cells stained with DAPI (Sigma, Saint Louis, MO, USA) were photographed for images of ZNF746, c-Myc, and DAPI by a Delta Vision imaging system (Applied Precision, Issaquah, WA, USA).

### 2.12. Statistical Analysis

All data represent means ± standard deviation (SD). For statistical analysis Student’s *t*-test was used for comparison of two groups by using GraphPad Prism software (Version 5.0, CA, USA). The statistical significance was determined at *p* value of < 0.05 between control and Morusin treated groups.

## 3. Results

### 3.1. Cytotoxic Effect of Morusin in Colorectal Cancer Cells

**Figure 1:**
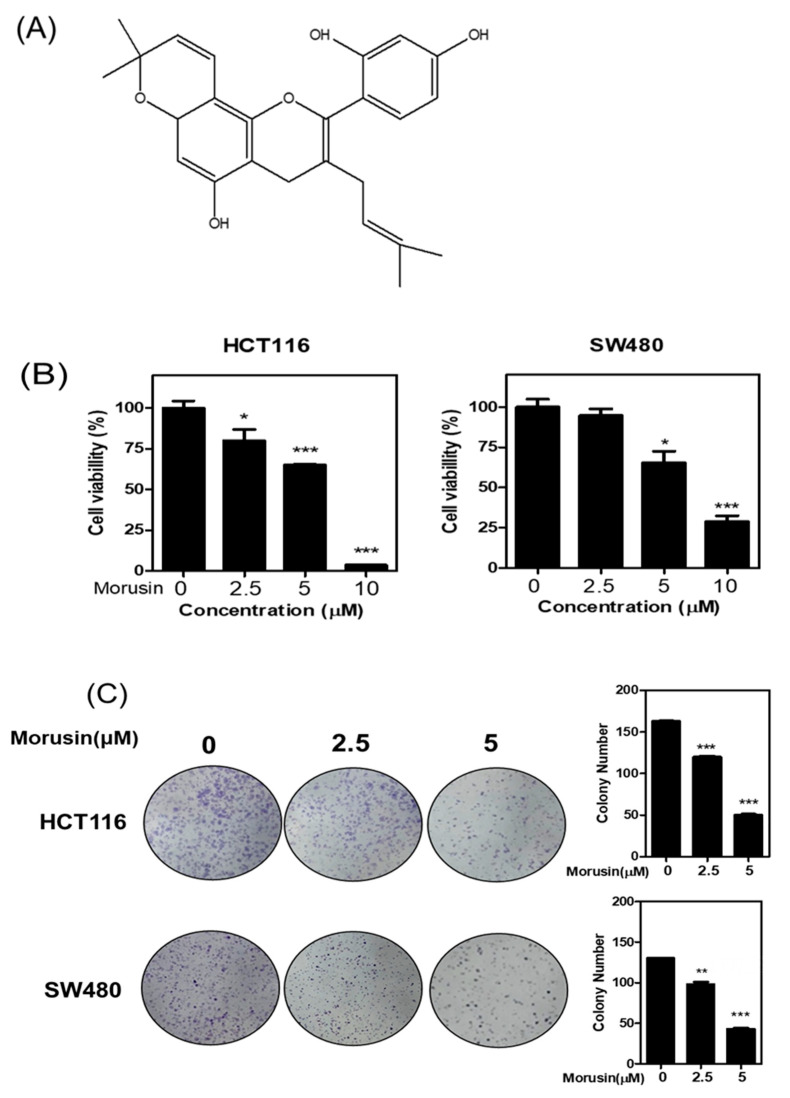
Effect of Morusin on cytotoxicity in HCT116 and SW480 cells. (**A**) Chemical structure of Morusin. (**B**) HCT116 and SW480 cells were exposed to various concentrations of Morusin (0, 2.5, 5, and 10 μM) for 24 h and cell viability was evaluated by MTT assay. Data stand for means ± SD of three independent experiments. * *p* < 0.05, ** *p* < 0.01 versus untreated control. (**C**) Photos for colony formation of Morusin (0, 2.5, and 5 μM) treated HCT116 and SW480 cells. The colonies were visualized by staining with Diff-Quick solution (Sysmex, Japan). Data represent means ± SD of three independent experiments. * *p* < 0.05, ** *p* < 0.01, *** *p* < 0.001 versus untreated control.

To explore the cytotoxic effect of Morusin (Figure 1A), a cell viability assay was carried out in HCT116 and SW480 CRCs by MTT assay. The cells were exposed to Morusin (0, 2.5, 5, and 10 μM) for 24 h. Here, Morusin inhibited the viability in HCT116 and SW480 cells (Figure 1B), while HCT116 cells were more susceptible to Morusin compared to SW480 cells. Likewise, Morusin reduced the number of colonies in HCT116 and SW480 cells by the colony formation assay (Figure 1C).

### 3.2. Morusin Induced Apoptosis in HCT116 and SW480 Cells

**Figure 2:**
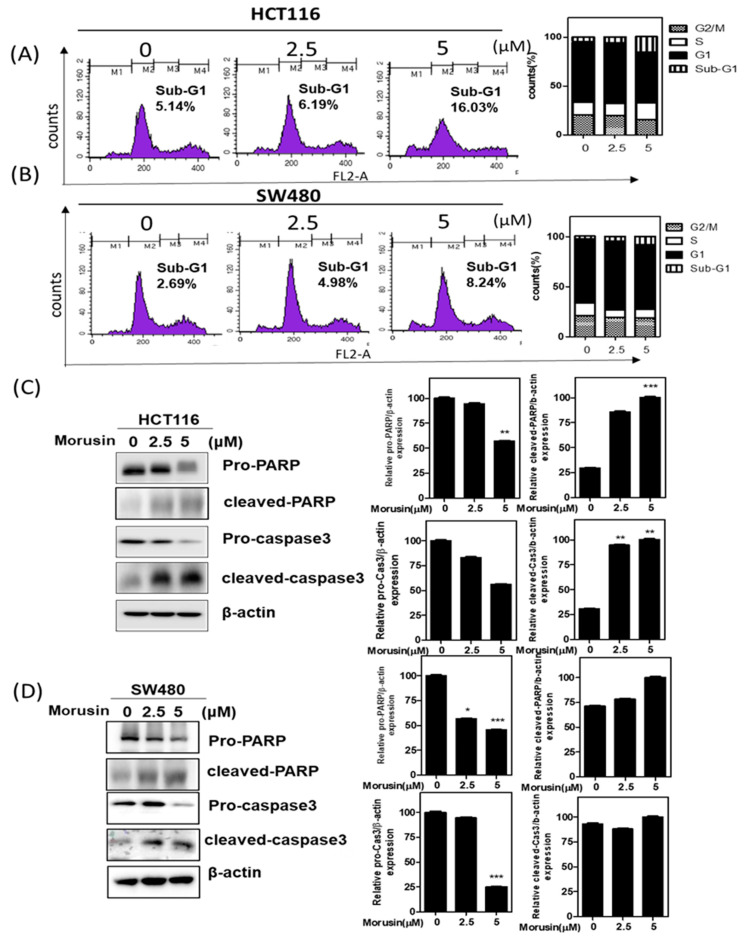
Effect of Morusin on apoptosis in HCT116 and SW480 cells. (**A**,**B**) Cell cycle analysis was conducted with propidium iodide (PI) staining by flow cytometry. Bar graphs showed quantification of cell cycle population (%) of three independent experiments. * *p* < 0.05, *** *p* < 0.001 vs. untreated control. (**C**,**D**) HCT116 and SW480 cells were exposed to Morusin for 24 h and subjected to Western blotting for PARP and caspase-3. Graphs stand for relative level of protein/β-Actin as means ± SD of three independent experiments. ** *p* < 0.01 and *** *p* < 0.001 versus untreated control.

To confirm the apoptotic effect of Morusin, cell cycle assay and Western blotting were conducted in Morusin treated HCT116 and SW480 cells. Herein, Morusin increased sub-G1 population in HCT116 and SW480 cells compared to untreated control (Figure 2A,B). Consistently, Morusin enhanced the cleavage of PARP, and reduced the expression of pro-PARP and pro-caspase 3 in HCT116 and SW480 cells (Figure 2C,D).

### 3.3. Morusin Attenuated the Expression of ZNF746 and c-Myc in HCT116 and SW480 Cells

**Figure 3:**
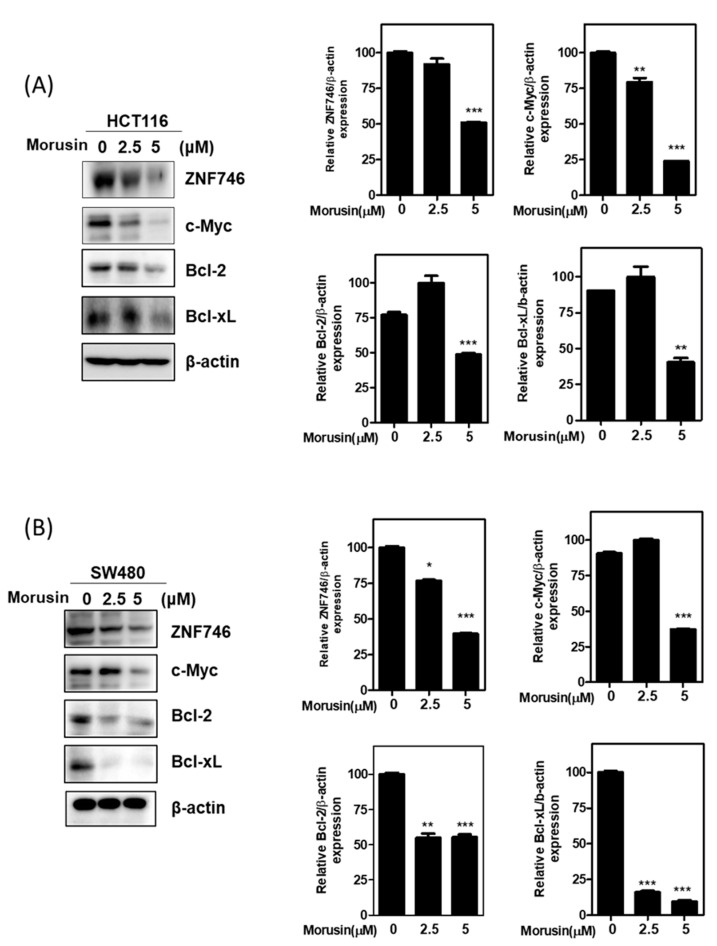
Effect of Morusin on the expression of ZNF746, c-Myc, Bcl-2, and Bcl-xL in HCT116 and SW480 cells. (**A**,**B**) Effect of Morusin on ZNF746, c-Myc, Bcl-2, and Bcl-xL in CRCs. HCT116 (**A**) or SW480 cells (**B**) were exposed to Morusin (0, 2.5, and 5 μM) for 24 h and subjected to Western blotting for ZNF746, c-Myc, Bcl-2, and Bcl-xL. Bar graphs stand for means ± SD for relative ZNF746, c-Myc, Bcl-2, and Bcl-xL protein expression. Data represent means ± SD of three independent experiments. * *p* < 0.05, ** *p* < 0.01 and *** *p* < 0.001 versus untreated control.

To determine the role of c-Myc and ZNF746 in Morusin induced apoptosis, Western blotting was performed in HCT116 and SW480 cells. As shown in Figure 3A,B, Morusin significantly attenuated the protein expression of c-Myc and ZNF746 in HCT116 and SW480 cells.

### 3.4. Morusin Reduced the Stability of ZNF746 and c-Myc in HCT116 and SW480 Cells in the Presence of Cycloheximide

**Figure 4:**
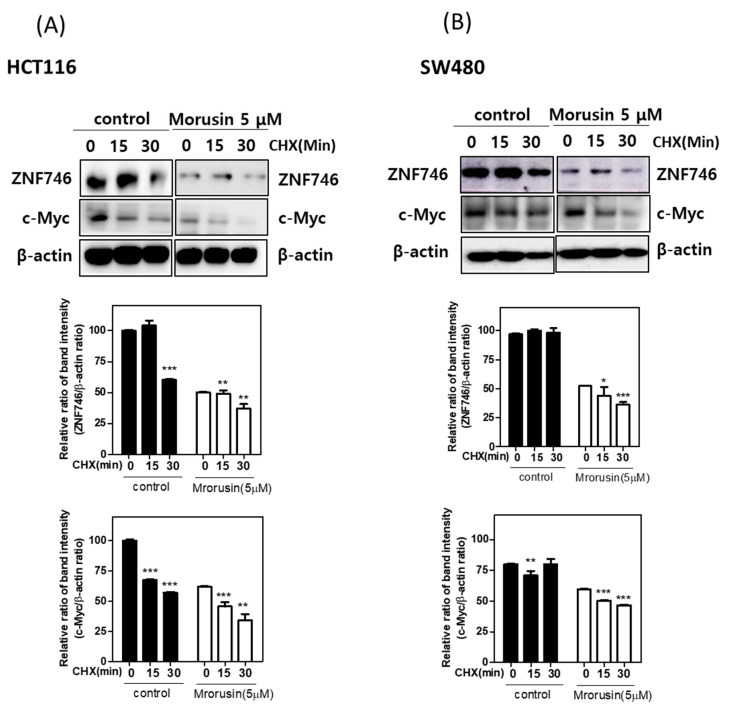
Effect of Morusin reduced the stability of ZNF746 and c-Myc in HCT116 and SW480 cells in the presence of cycloheximide. (**A**,**B**) Effect of cycloheximide on the expression of ZNF746 and c-Myc in Morusin treated HCT116 and SW480 cells. Assay was conducted in independent triplicates. * *p* < 0.05, ** *p* < 0.01 and *** *p* < 0.001 versus untreated control.

To confirm whether Morusin regulates the stability of ZNF746 and c-Myc, a cycloheximide assay was carried out in HCT116 and SW480 cells. As shown in Figure 4A,B, Morusin reduced the half-life stability of ZNF746 and c-Myc from 15 min in HCT116 or SW480 cells exposed to DNA synthesis inhibitor cycloheximide.

### 3.5. Ectopic Expression of ZNF746 Reduces Apoptotic Effect of Morusin in HCT116 Cells

**Figure 5:**
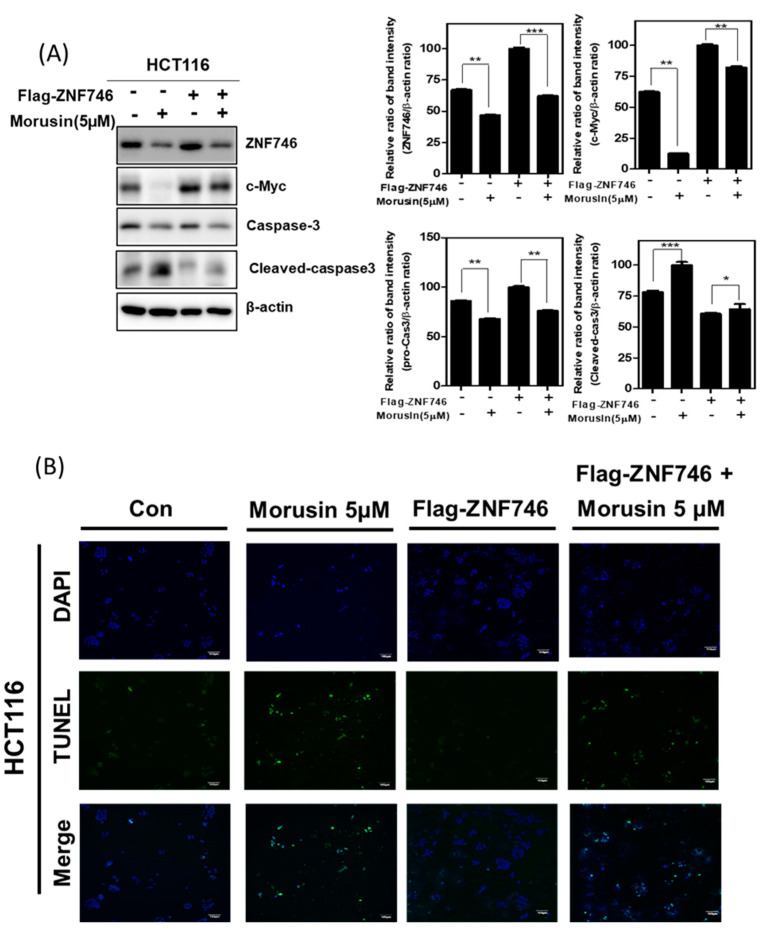
Ectopic expression of ZNF746 reduced apoptotic effect of Morusin in HCT116 cells. (**A**) Effect of ZNF746 overexpression on c-Myc and caspase-3 in HCT116 cells. (**B**) Effect of ZNF746 overexpression on the number of TUNEL-positive cells in HCT116 cells. The fluorescent signals from fragmented DNA (green), and DAPI (blue) by FLUOVIEW FV10i confocal microscopy. Magnification bar = 100 µm. * *p* < 0.05, ** *p* < 0.01 and *** *p* < 0.001 vs. untreated control. Assay was conducted in independent triplicates.

To confirm the important role of ZNF746, Western blotting and TUNEL assays were carried out in HCT116 cells transfected with overexpression plasmid of ZNF746. As shown in Figure 5A,B, overexpression of ZNF746 reversed the ability of Morusin to attenuate the expression of c-Myc and ZNF746 and the number of TUNEL positive cells in HCT116 cells.

### 3.6. Morusin Disrupted the Binding of c-Myc and ZNF746 in HCT116 Cells

**Figure 6:**
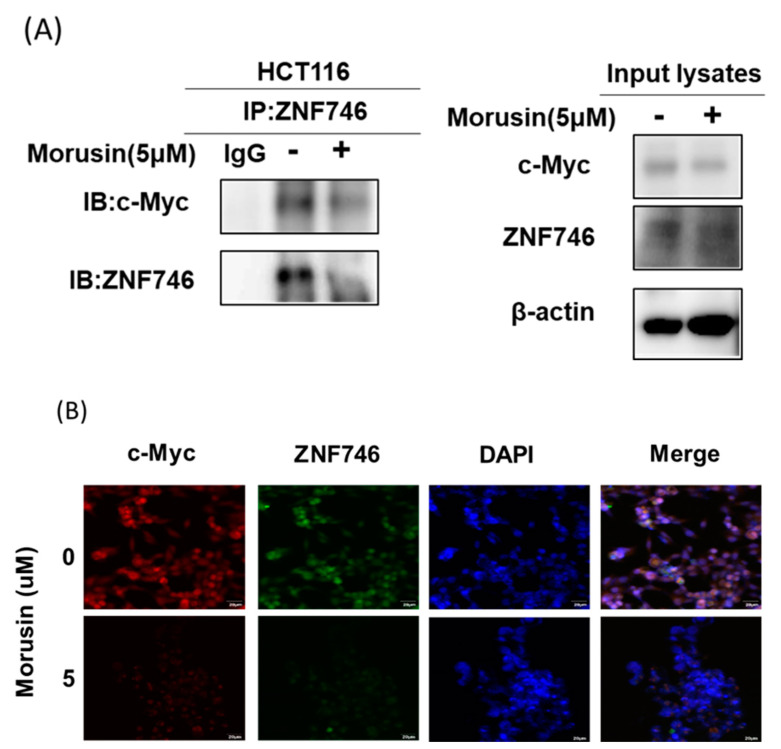
Effect of Morusin on interaction between c-Myc and ZNF746 in HCT116 cells by immunoprecipitation and immunofluorescence. (**A**) Effect of Morusin on the binding of c-Myc and ZNF746 in HCT116 cells by immunoprecipitation. (**B**) Effect of Morusin on the colocalization between c-Myc and ZNF746 in HCT116 cells by Immunofluorescence. Scale bars = 20 μm.

To confirm whether Morusin disrupts interaction between ZNF746 and c-Myc, immunoprecipitation was carried out in Morusin treated HCT116 cells. Here, Morusin suppressed the binding of c-Myc and ZNF746 in HCT116 cells (Figure 6A). Consistently, immunofluorescence reveals that c-Myc (red) was completely merged to ZNF746 (green) in HCT116 cells (Figure 6B).

### 3.7. miR193a-5p Plays a Pivotal Role in Morusin-Induced Apoptosis in HCT116 Cells

**Figure 7:**
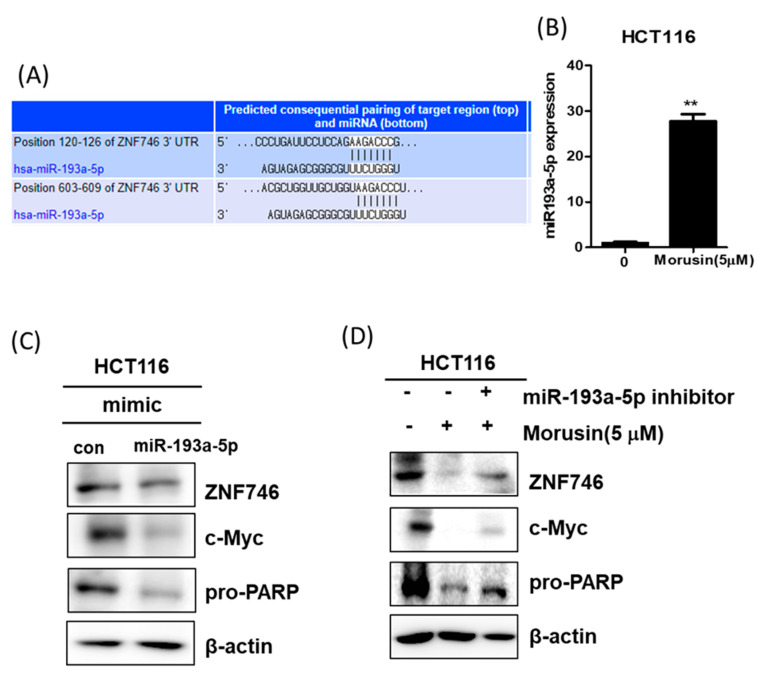
Effect of miR-193a-5p mimic or inhibitor in Morusin-induced apoptosis in HCT116 cells. (**A**) TargetScan web server predicts the binding sequence of miR-193a-5p in the 3′-UTR of ZNF746 and c-Myc. (**B**) Effect of Morusin on the expression of miR193a-5p in HCT116 cells by qRT-PCR. Assay was conducted in independent triplicates. ** *p* < 0.01 versus untreated control. (**C**) Effect of miR193a-5p mimic on ZNF746, c-Myc, and pro-PARP in HCT116 and cells. (**D**) Effect of miR193a-5p inhibitor on ZNF746, c-Myc, and pro-PARP in HCT116 cells.

To determine the role of miR193a-5p in Morusin-induced apoptosis, Western blotting was performed in HCT116 cells. Interestingly, TargetScan web server predicts that miR-193a-5p directly binds to the 3′-Untranslated region of c-Myc and ZNF746 by bioinformatics analysis (Figure 7A). Additionally, Morusin increased the mRNA expression of miR193a-5p in HCT116 cells (Figure 7B). Furthermore, miR193a-5p mimic reduced the expression of ZNF746, c-Myc, and pro-PARP in HCT116 cells (Figure 7C). In contrast, miR193a-5p inhibitor masked the ability of Morusin to suppress the expression of ZNF746, c-Myc, and pro-PARP in HCT116 cells (Figure 7D), implying the partial role of miR193a-5p in Morusin-induced apoptosis.

## 4. Discussion

Recently, several natural compounds, including phenolic compounds, phytosterols, nitrogen compounds, carotenoids, iridoids, organosulfur compounds, essential oils, and dietary fibers [27], are gaining interest due to significant antitumor effects and low toxicity in colorectal cancers (CRCs) with combination therapy potential [28]. Though previous evidence reveals that Morusin suppresses the growth of colorectal cancer stem-like cells [3] and induces apoptosis in HT-29 CRCs via inhibition of NF-kB [29], the underlying antitumor mechanism of Morusin still remains unclear to date. Thus, in the current work, the antitumor mechanism of Morusin was investigated in association with C-Myc and ZNF746 mediated by miR193a-5p in HCT116 and SW480 cells. Herein, Morusin reduced the viability and the number of colonies in HCT116 and SW480 cells, implying the cytotoxic and anti-proliferative effect of Morusin. It is well known that subG1 accumulation represents apoptosis in the cells [30], and cleavages of PARP and caspases indicate intrinsic or extrinsic apoptosis [31,32,33]. Consistently, Morusin increased sub-G1 population, and cleaved PARP and caspase-3 in HCT116 and SW480 cells, indicating the apoptotic effect of Morusin in CRCs.

Emerging evidence indicates that c-Myc is a known nuclear transcription factor oncogene among the Myc family, comprising of n-Myc, c-Myc, and l-Myc, in several cancers [34,35]. Thus, c-Myc inhibitors are considered to control tumor initiation and progression [36,37]. Additionally, ZNF746 is known to promote cancer progression via c-Myc stability in CRC [1], bladder cancer [38], and lung cancer [12]. Herein, Morusin inhibited the expression of c-Myc and ZNF746, and the stability of c-Myc, and disrupted the direct binding through nuclear colocalization in HCT116 cells. Conversely, overexpression of ZNF746 masked the antitumor effect of Morusin to reduce the expression of c-Myc and increase the number of TUNEL positive cells in HCT116 cells, demonstrating the pivotal role of C-Myc and ZNF746 in Morusin induced apoptosis.

Accumulating evidence reveals that miRNAs are critically involved in the development, cell differentiation, cell cycle, apoptosis, metastasis, and angiogenesis in several cancers as an oncogene or tumor suppressor [39,40]. Among several miRNAs, miR193a-5p is reported to inhibit liver carcinogenesis [41] and induce G1 arrest in CRCs [42]. RT-PCR reveals that Morusin upregulated miR193a-5p in HCT116 cells. As shown in bioinformatics data, miR-193a-5p directly binds to the 3′-Untranslated region of ZNF746 and c-Myc; miR193a-5p mimic reduced the expression of ZNF746, c-Myc, and pro-PARP in HCT116 cells, while miR193a-5p inhibitor reduced the ability of Morusin to suppress the expression of ZNF746, c-Myc, and pro-PARP in HCT116 cells, indicating miR-193a-5p suppresses the expression of ZNF746, c-Myc, and pro-PARP during apoptotic effect of Morusin.

## 5. Conclusions

**Figure 8:**
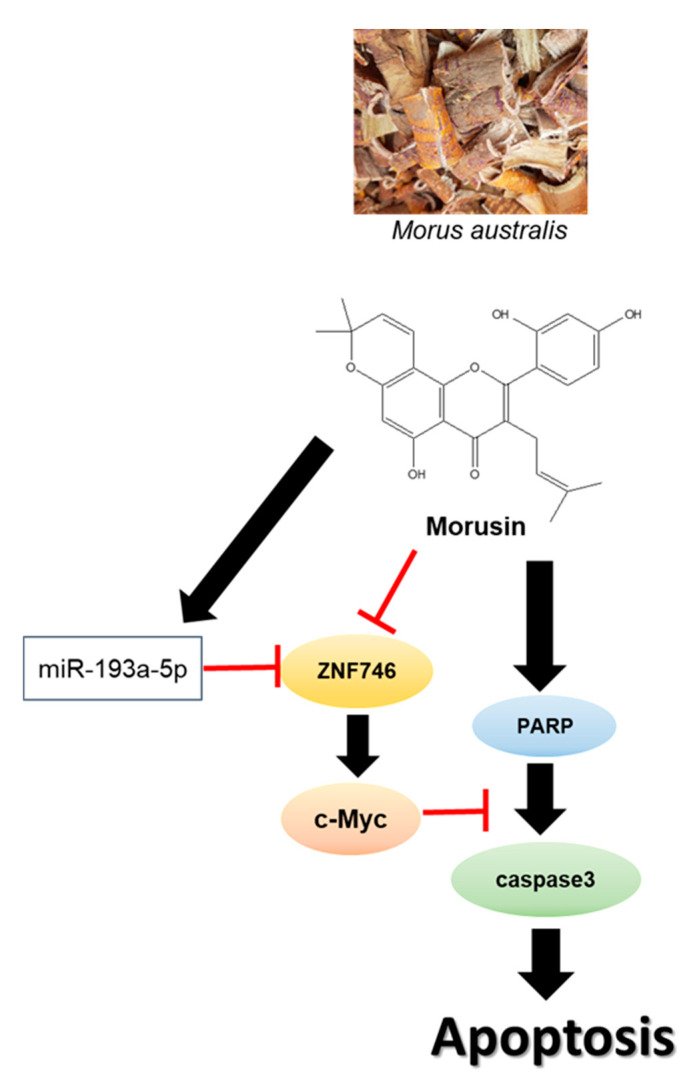
Schematic diagram on the apoptotic mechanism of Morusin via miR193a-5p mediated inhibition of C-Myc and ZNF746 signaling in CRCs.

Taken together, Morusin increased cytotoxicity and sub G1 population, cleaved PARP, and attenuated the expression of pro-caspase-3 and pro-PARP in HCT116 and SW480 cells. Additionally, Morusin suppressed the expression of C-Myc and ZNF746, disturbed the binding of ZNF746 and c-Myc, and upregulated miR193a-5p in HCT116 cells. Conversely, the miR193a-5p inhibitor masked the ability of Morusin to attenuate the expression of ZNF746, c-Myc, and pro-PARP in HCT116 cells. Overall, these findings suggest that miR193a-5p mediated inhibition of C-Myc and ZNF746 signaling plays a pivotal role in Morusin induced apoptosis in CRCs (Figure 8).
